# Diagnosis and Treatment of Small Vessel Childhood Primary Angiitis of the Central Nervous System (sv-cPACNS): An International Survey

**DOI:** 10.3389/fped.2021.756612

**Published:** 2021-10-12

**Authors:** Paul Keenan, Jürgen Brunner, Angela S. Quan, Martin Smitka, Gabriele Hahn, Clare E. Pain, Renate Häfner, Fabian Speth, Lucia Gerstl, Christian M. Hedrich

**Affiliations:** ^1^Department of Women's and Children's Health, Institute of Translational Medicine, University of Liverpool, Liverpool, United Kingdom; ^2^Pädiatrische Rheumatologie, Department Kinder- und Jugendheilkunde, Medizinische Universität Innsbruck, Innsbruck, Austria; ^3^Klinik und Poliklinik fur Kinder- und Jugendmedizin, Universitätsklinikum Carl Gustav Carus, Technische Universität Dresden, Dresden, Germany; ^4^Institut und Poliklinik für Radiologie, Universitätsklinikum Carl Gustav Carus, Technische Universität Dresden, Dresden, Germany; ^5^Department of Paediatric Rheumatology, Alder Hey Children's NHS Foundation Trust Hospital, Liverpool, United Kingdom; ^6^Deutsches Zentrum für Kinder- und Jugendrheumatologie, Garmisch-Partenkirchen, Germany; ^7^Zentrum für Geburtshilfe, Kinder- und Jugendmedizin, Klinik und Poliklinik für Kinder- und Jugendmedizin, Universitätsklinikum Eppendorf, Hamburg, Germany; ^8^Division of Paediatric Neurology, Developmental Medicine and Social Paediatrics, Department of Paediatrics, Dr. von Hauner Children's Hospital, Ludwig-Maximilians-University Munich, Munich, Germany

**Keywords:** vasculitis, CNS, childhood, pediatric, treatment, diagnosis, inflammation, CNS inflammation

## Abstract

Childhood primary angiitis of the Central Nervous System (cPACNS) is a rare autoimmune and inflammatory disease. It can result in significant neuronal damage, neurodevelopmental delay and potentially death. Childhood PACNS is divided into subcategories: angiography-positive p-cPACNS that affects medium and large vessels, and angiography-negative small vessel sv-cPACNS. Due to its rarity, variable clinical representation, and the lack of a diagnostic criteria and therapeutic plans, diagnosis and treatment of cPACNS is challenging and approaches vary. This survey collected information on diagnostic and therapeutic approaches to sv-PACNS. It was shared with international clinician networks, including the German Society for Paediatric Rheumatology, the Paediatric Rheumatology European Society, the “Network Paediatric Stroke,” and members of the American College of Rheumatology/CARRA Paediatric Rheumatology list server. This project has shown consensus in numerous diagnostic and therapeutic treatment approaches, highlighting key areas which will be utilised to develop statements in the use of expert consensus meetings to standardise diagnostic and therapeutic approaches in this rare inflammatory disease.

## Introduction

Childhood primary angiitis of the central nervous system (cPACNS) is a rare inflammatory condition that can result in significant neuronal damage, neurodevelopmental delay, and potentially be life-threatening (1, 2). Based on vessels size affected, it can be subdivided into (i) angiography positive large-medium vessel vasculitis (p-cPACNS) and (ii) angiography negative small vessel vasculitis (sv-cPACNS) (2–5). Diagnosis and timely treatment induction can be complicated by limited awareness and reservations in relation to invasive diagnostics (brain biopsies) even among specialists and (highly) variable clinical features that can be affected by individual factors, pre-existing medical conditions, the size and region of vessels affected, etc. Diffuse symptoms of cPACNS can include behavioural changes, psychiatric problems, slowly or rapidly developing headaches, cognitive decline and/or impairment, slowly progressing or acute neurologic defects (as in ischemic stroke), seizures, and others (6). Calabrese et al. (7) provided clinical criteria to diagnose adults with PACNS that include (1) an unexplained neurological deficit, (2) histological/angiographic inflammation within the CNS, and (3) the lack of another condition in the patient which could explain these features. Benseler et al. (8) modified criteria for the use in children and young people by including an additional criterion: (4) recently developed psychiatric or neurological defects.

While the current understanding of cPACNS molecular pathophysiology is limited, inflammation of vessel walls is the hallmark feature. As in sv-cPACNS, inflammation is limited to small vessels; magnetic resonance imaging (MRI) and conventional angiography fail to detect vessel wall inflammation (3, 9). However, indirect signs of inflammation and resulting ischemia, leptomeningeal enhancement, and signs of impaired blood-brain barrier function can be detected (10). In sv-cPACNS, analysis of the cerebrospinal fluid delivers increased CSF protein concentrations alongside pleocytosis in the majority of cases. Furthermore, inflammatory CSF cytokine signatures, and in some cases (up to 50%) increased systemic blood inflammatory markers have been reported (11). As none of the aforementioned findings are specific for sv-cPACNS, brain biopsy is considered to be the only definitive diagnostic test for sv-cPACNS, and criteria for sv-cPACNS include signs of vasculitis with perivascular lymphocyte infiltrates on brain biopsy as a necessary requirement for classification (11, 12).

Currently, no clinical trials, licenced treatments or evidence-based recommendations exist for cPACNS. Thus, treatment is empiric and, in addition to controlling/inhibiting coagulation aims at rapid and sustained inhibition of inflammation. Independent of vessel-sizes affected, anti-inflammatory induction treatment usually includes corticosteroids, and some colleagues concomitantly administer cyclophosphamide (12, 13). In most forms of PACNS, including sv-PACNS, maintenance treatment with disease-modifying antirheumatic drugs (DMARDs) aims at preventing disease flares and relapses (12, 13). In adult PACNS patients, Salvarani et al. (14) demonstrated that systemic corticosteroids combined with the DMARD mycophenolate mofetil (MMF) can be effective and less toxic when compared to cyclophosphamide induction treatment. A manuscript reporting a small cohort of 5 patients with p-cPACNS, recently suggested the same in children (15). However, published reports on induction and maintenance treatment in sv-cPACNS is even more limited (16).

To harmonise and optimise diagnosis, treatment and outcomes, consensus treatment plans (CTP) can be a tool to generate evidence in rare conditions where clinical trials are currently not available and/or achievable. They allow prospective data collection in patient cohorts receiving one available treatment choice therapy. Thus, treatment responses and outcomes can be associated with standard of care approaches. The objective of this survey was to collect information from international experts on diagnostic and therapeutic approaches in sv-cPACNS. This study is closely related to a previous survey on p-cPACNS (17), and collected information on standard of care approaches to diagnosis and treatment in sv-cPACNS to, in a next step, generate statements that can then be used towards an expert agreement for CTP development (18, 19).

## Methods

A survey was designed to collect information on diagnostic and therapeutic approaches in sv-cPACNS by experts in the field and was closely related to a previously published survey focusing on p-cPACNS (17). The survey was based on real-life cases treated at the Department of Paediatrics, Faculty of Medicine Carl Gustav Carus, University of Technology, Dresden, Germany. The survey was developed by the authors (namely J.B., M.S., G.H., R.H., F.S., L.G., and C.M.H) and shared among colleagues from Alder Hey NHS Children's Foundation Trust Departments of Rheumatology and Neurology for beta testing; input from colleagues was integrated into the final survey. It consisted of introductory questions addressing demographics (sub-specialty, country of practise) and experience of participants, and a representative case scenario accompanied by multiple choice questions (Appendix in Supplementary Material). Case based questions aimed at determining examinations deemed important to diagnose sv-cPACNS. Furthermore, respondents were asked which specialties they consider important to be involved in the diagnosis and treatment of sv-cPACNS patients. Multiple choice answers were provided with respondents being able to select one or multiple options at the same time as indicated (Appendix in Supplementary Material), as well as the option to add comments and/or additional answers.

The survey was conducted online using SurveyMonkey (www.surveymonkey.com). The survey was shared with international colleagues with experience in the diagnosis and treatment of sv-cPACNS at the same time to ensure no duplicate responses. Furthermore, responses were checked for similarity of demographic information and responses during analysis to exclude potential duplicates (but none were identified). It was open for 2 months with reminder emails sent at 4 and 6 weeks. The email addresses used in this survey are collected from the German Society for Paediatric Rheumatology (GKJR) (*n* = 151; Paediatric Rheumatologists; personal email), members of the German-speaking “Paediatric Stroke Group” with members in Germany, Switzerland, and Austria (*n* = 72; including Paediatric Rheumatologists, Immunologists, Neurologists, and Haematologists; personal email), the Paediatric Rheumatology European Society (PRES) (*n* = 7800; society members; monthly PRES email newsletter) and subscribers to the American College of Rheumatology/CARRA Paediatric Rheumatology Bulletin Board (ped-rhe-list-bounces@mcmaster.ca) (*n* = 1849; personal email).

Descriptive analysis of responses was performed using Microsoft Excel (Redmond, Washington, USA). To adjust for incomplete responses, for each individual question, the number of responses were provided across this manuscript, and percentages were calculated based on the number of responses to each individual question of the survey.

## Results

### Respondents' Demographics and Experience

**Figure 1:**
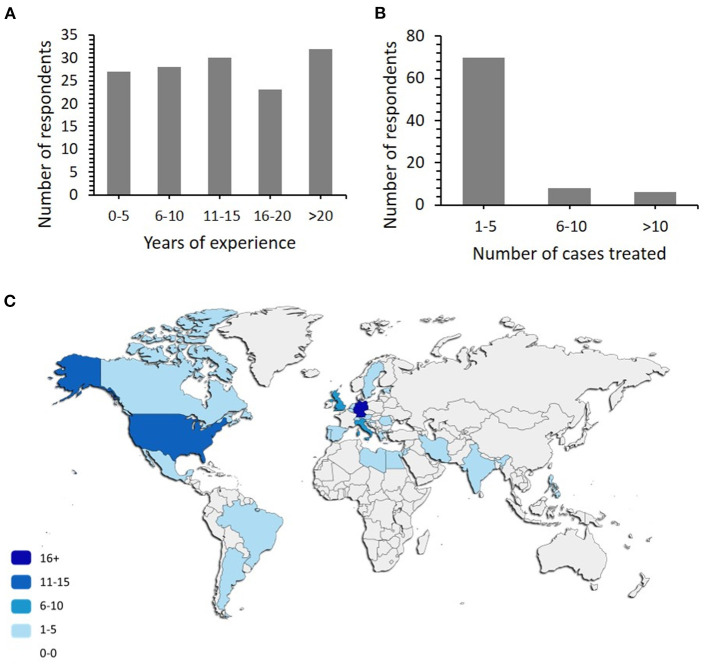
Demographics of participants. **(A)** Portrays the number of years' experience respondents have working in their speciality. **(B)** Number of patients with sv-cPACNS treated. To respondents answered treating more than 20 and 80 cases, respectively. **(C)** Map depicting the countries the respondents work in. This is done using a shading scale and legend. The darker the shading indicates a higher number or respondents from that country.

The survey was answered by a total of 140 clinicians, an overwhelming majority specialised in paediatric rheumatology (110; 78.6%), followed by paediatric neurology (18; 12.8%), general paediatrics (6), and haematology (4). One adult rheumatologist and one adult neurologist also participated in the survey (Q1; *N* = 140 responses). Years of experience of responders in their individual specialities (Q2, *N* = 140 responses) and the number of sv-cPACNS treated varied, with 27/140 having 0-5 years' experience, 28/140 had 6–10, 30/140 had 16–20 with 32/140 respondents having over 20 years' experience in their speciality. In terms of their experience treating sv-cPACNS patients (Q4, *N* = 140 responses), 47/140 respondents had no personal experience with the diagnosis and treatment of sv-cPACNS (Figure 1A: Q2, Figure 1B: Q4). The majority of responses were received from Europe and North America (Figure 1C: Q3, *N* = 133 responses), represented by Germany *N* = 54, USA *N* = 15, Italy *N* = 8, UK *N* = 7, Brazil, Canada, and Turkey *N* = 5, Greece and Sweden *N* = 4, Netherlands and Croatia *N* = 3, Portugal, Mexico, Spain and Egypt *N* = 2, with Iran, Argentina, Jordan, India, Estonia, Romania, Switzerland, Belgium, Philippines, Libya, and Austria all having *N* = 1.

### Case Study

After aforementioned general questions, the survey presented a clinical scenario addressing specific diagnostic and therapeutic approaches in a paediatric patient with new neurological defects: *What would you do with the following clinical scenarios? A previously healthy 10-year-old girl came to hospital with an* ~*1 month-long history of headaches, nausea, and gradual hemiparesis on her left. She had been admitted 6 weeks previously with left-sided focal seizures*.

### Diagnostic Approach

Participants were asked whether they thought genetic testing plays a role in diagnosing sv-cPACNS; 60/116 responders (51.72%) suggested that genetic testing should be performed (Q5, *N* = 116 responses). Of the 49 respondents who further specified, 32 (65%) suggested genetic testing for DADA2 (*CECR1* mutations), 5 suggested genetic testing for mutations in *COL4A1* (Gould syndrome). Other suggestions included testing for CADASIL (Cerebral Autosomal Dominant Arteriopathy with Sub-cortical Infarcts and Leukoencephalopathy), performing a primary immunodeficiency panel with immune regulatory and immune deficiency genes, monogenic autoinflammation diseases, Chronic Atypical Neutrophilic Dermatosis with Lipodystrophy and Elevated temperature (CANDLE).

Based upon previously mentioned clinical information, participants were asked to select the 5 most likely differential diagnoses (Q6, *N* = 120 responses). Multiple responses were possible. Most respondents selected CNS vasculitis as the most likely differential diagnosis (95%), closely followed by tumour (90%), Ischemic stroke (63.33%), infection, e.g., meningitis (60.83%), and multiple sclerosis (50%) (Table 1).

**Table 1:** Diagnostic approach to sv-cPACNS.

**5 most likely differential diagnosis (Q6)**			
1.	CNS Vasculitis	114/120	95.00%
2.	Tumour	108/120	90.00%
3.	Ischemic stroke	76/120	63.33%
4.	Infection, e.g., meningitis	73/120	60.83%
5.	Multiple sclerosis	60/120	50.00%
6.	CNS Tuberculosis	37/120	30.83%
7.	Migraine	34/120	28.33%
8.	Traumatic intracranial bleeding	25/120	20.83%
9.	Congenital anatomical anomaly	24/120	20.00%
10.	Funicular myelose	4/120	3.33%
11.	Others, included associated encephalitis, lymphoma, prolonged non-traumatic bleeding, neuronal antibody-mediated diseases, as well as other paediatric neurological diseases	19/120	15.83%
**What first-line investigations would you perform? (Q7)**			
1.	Blood tests (including full blood counts, inflammatory markers, and clotting tests)	107/120	89.17%
2.	Emergency MRI of the brain including angio-MRI	103/120	85.83%
3.	Lumbar puncture and CSF analysis, including cell counts and differentiation, protein, lactate, glucose, microbial cultures, herpes virus PCR, VZV PCR, Borrelia IgG, IgM	93/120	77.50%
4.	Brain CT scan including CT-angiography	29/120	24.17%
5.	None	0/120	0.00%
6.	Other, included 8 ECG/EEG, borrelia classification, lab studies for lupus and autoimmune diseases as well as CSF tests for IgG and oligoclonal bands, with another suggesting the testing for ANCA, ANAs antibodies	18/120	15.00%
**If you chose blood tests, which of the following bloods would you order? (Q8)**			
1.	Full blood count (including complete white cell count)	111/115	96.52%
2.	Immunology (e.g., ANA, ENA, Complement Factors, Cardiolipin AB, ANCA)	106/115	92.17%
3.	Clotting tests (including PTT, INR, fibrinogen, D dimers)	104/115	90.43%
4.	Interferon Gamma Release Assay Test (for TB infection)	55/115	47.83%
5.	Adenosine Deaminase 2 Activity (ADA2)	55/115	47.83%
6.	None	0/115	0.00%
7.	Others, included acute phase reactants (CRP, ESR), immunoglobulins such as Borrelia, vWF, IL-2, lupus anticoagulant, ferritin, in the instance of ADA2 clarification, MRI and other forms of imaging, neuronal antibodies, liver and kidney values along with tests for infectious disease	22/112	19.13%
**If you chose to look at blood immunology, which of the following would you look at? (Q9)**			
1.	Anti-phospholipid Antibodies/Anti-Phospholipid	106/113	93.81%
2.	Antinuclear antibodies (20)	103/113	91.15%
3.	Anti-Neutrophil Cytoplasmic Antibodies (ANCA)	100/113	88.50%
4.	Anti-double stranded DNA (dsDNA)	98/113	86.73%
5.	Complement factors and complement cascade activation	91/113	80.53%
6.	Anti-NMDA and Aquaporin Antibody	77/113	68.14%
7.	None	0/113	0.00%
8.	Others, included Extractable nuclear antigen, rheumatoid Factor (18), Thyroid Peroxidase (TPO) antibodies, Anti-Aquaporin-4 (Anti-AQP4) antibody, Anti-Ro, anti-La, anti-RNP, Other JSLE antibodies associated with neuro SLE-ribosomal P, NMDA, LAC as well a APL antibodies, Another respondent also suggested testing for interleukin 6	14/113	12.39%
**If you chose lumbar puncture, which of the following would you look at? (Q10)**			
1.	Cell count and differentiation	108/114	94.74%
2.	Protein	108/114	94.74%
3.	Oligoclonal bands	102/114	89.47%
4.	Glucose	94/114	82.46%
5.	Microbial cultures	92/114	80.70%
6.	CSF opening pressure	88/114	77.19%
7.	Anti-NMDA and Aquaporin Antibodies	79/114	69.30%
8.	Lactate	75/114	65.79%
9.	No cerebrospinal fluid tests	0/114	0.00%
10.	Others indicated they would determine viral PCR information (i.e., HSV, echovirus, enterovirus). Other parameters determined included neuronal biomarkers such as neopterin and biopterin levels as well as neuronal antibodies such as anti-MOG, Aquaporin, Intrathecal IgG synthesis, anti-NMDA receptor antibodies, and Myelin basic protein antibodies, with other respondents suggesting other parameters determined were dependant on imaging. Two respondents indicated that acid-fast bacilli would be determined as well as further immunological clarification into CERC1 deficiency was required	25/114	21.93%

Next, participants were asked what first line of investigations they would perform (Q7, *N* = 120 responses). The majority of respondents indicated that initial investigations should include blood tests (including full blood counts, inflammatory markers and clotting tests) (89.17%), followed by emergency MRI of the brain including angio-MRI (85.83%), lumbar puncture and CSF analysis, including cell counts and differentiation, protein, lactate, glucose, microbial cultures, herpes virus PCR, VZV PCR, Borrelia IgG, IgM (77.50%), 24.17% suggested performing a Brain CT scan including CT- angiography (Table 1).

When asked what blood laboratory tests to consider (Q8, *N* = 115 responses), 96.5% suggested full blood count (including complete white cell count), followed by Immunology (e.g., antinuclear antibodies (ANA), ENA, Complement Factors, Cardiolipin antibody, Anti-Neutrophil Cytoplasmic Antibodies (ANCA), (92.17%), clotting tests (including PTT, INR, fibrinogen, D dimers) (90.43%), Interferon Gamma Release Assays (for TB infection) and Adenosine Deaminase 2 Activity (ADA2) (each 47.83%). Specifying immunological laboratory tests (Q9, *N* = 113 responses), anti-phospholipid antibody tests were most frequently selected (93.81%), followed by ANA (20) (91.15%), ANCA (88.50%), Anti-double stranded DNA antibodies (dsDNA) (86.73%), complement factors and complement cascade activation (80.53%), and Anti-NMDA and Aquaporin Antibodies (68.14%). All participants agreed that immunological laboratory tests were required. Considering CSF tests (Q10, *N* = 114 responses), cell counts and differentiation along with protein levels were most frequently requested (94.74% each), followed by oligoclonal bands (89.47%), glucose levels (82.46%), microbial cultures (80.70%), CSF opening pressure (77.19%), Anti-NMDA and Aquaporin Antibodies (69.30%), and lactate levels (65.79%). All participants agreed that CSF testing was required (Table 1).

Participants were asked which emergency MRI sequences they consider of interest and helpful (Q11, *N* = 114 responses). Responses varied considerably with most respondents requesting MR angiography (86.84%), followed by FLAIR (Fluid-attenuated inversion recovery) (74.56%), Diffusion-weighted MRI (20) (66.67%), T1 FS, contract enhanced (54.39%), T2 with fat saturation (FS) (46.49%), T1 FS (35.96%), TIRM (Turbo inversion recovery magnitude)/STIR (Short tau inversion recovery) (34.21%). One participant suggested that no MRI was required (Table 2).

**Table 2:** Diagnostic approach continued.

**If you chose Emergency MRI, which of the following are you interested in? ASAP, same day (Q11)**			
1.	MR Angiography	99/114	86.84%
2.	FLAIR (Fluid-attenuated inversion recovery)	85/114	74.56%
3.	Diffusion-weighted MRI (20)	76/114	66.67%
4.	T1 FS, contract enhanced	62/114	54.39%
5.	T2 FS	53/114	46.49%
6.	T1 with fat saturation (FS)	41/114	35.96%
7.	TIRM (Turbo inversion recovery magnitude)/STIR (Short tau inversion recovery)	39/114	34.21%
8.	No MRI required	1/114	0.88%
9.	Others, most respondents who selected other suggested that they would consult radiologists for this aspect of diagnosis, while in an emergency a CT would be done. Otherwise, all sequences are routinely done here	19/114	16.67%
**The diagnosis suspected small vessel vasculitis is made. Would you perform a brain biopsy to confirm the diagnosis? (Q12)**			
	Yes	53/115	46.09%
	No	61/115	53.04%
	If not, what would you do instead? Some respondents suggested that a brain biopsy is usually too invasive and are unlikely to persuade neurosurgeons to perform leading the to treat multiple pathologies together. However, some respondents also indicated that theoretically this should be done to nail down diagnosis although this may be dangerous and should only be performed if lesions are accessible and diagnosis is unclear	41/115	
**In which of these situations would you perform a brain biopsy? (Q13)**			
1.	A patient on ICU in status epilepticus, ventilated, with aforementioned MRI, and lesions are accessible to biopsy	79/111	71.17%
2.	Otherwise, stable patient with slowly progressing hemiplegia and aforementioned MRI findings	65/111	58.56%
3.	Patient in ICU in status epilepticus, ventilated, with aforementioned MRI, and lesions not accessible for biopsy (-> biopsy from non-lesional tissue)	17/111	15.32%
4.	Never	13/111	11.71%
5.	Patient on ICU in status epilepticus, ventilated, with no findings on MRI	11/111	9.91%

Next, additional clinical information was provided: *Cerebrospinal fluid opening pressure, cell count, cytology, protein, and glycose concentrations were normal, and cultures and HSV polymerase chain reaction were negative. On brain MRI (FLAIR sequences), signal alterations in the parietal region were identified. T1 gadolinium enhanced sequences unveiled enhancement in the affected region*.

Based on new information, the diagnosis of suspected sv-cPACNS was made. Participants were asked whether they consider a brain biopsy to secure the diagnosis, (Q12, *N* = 114 responses). Multiple options could be chosen. Responses were almost equally split, with 53.51% suggesting a brain biopsy was not required, and 46.49% arguing for a brain biopsy being required to secure diagnosis. Next, participants were asked under which clinical circumstances they would perform a brain biopsy (Q13, *N* = 111 responses). Most respondents would perform brain biopsy in “a patient on ICU in status epilepticus, ventilated, with aforementioned MRI, and lesions that are accessible to biopsy” (71.17%), followed by “an otherwise stable patient with slowly progressing hemiplegia and aforementioned MRI findings” (58.56%), a “patient on ICU in status epilepticus, ventilated, with aforementioned MRI, and lesions not accessible for biopsy (biopsy from non-lesional tissue)” (15.32%), and “a patient on ICU in status epilepticus, ventilated, with no findings on MRI” (9.91%). Notably, 11.71% of respondents suggested that a brain biopsy should never be performed in the clinical scenario presented (Table 2).

### Therapeutic Approach

Next, participants were informed that: *Brain biopsy showed a small vessel, non-granulomatous, non-necrotic, lymphocytic angiitis. You have made the diagnosis of primary small vessel vasculitis based on clinical findings, lab and MRI findings, plus suggestive biopsy*.

Participants were asked which treatment they would choose in the above reported patient given all clinical and laboratory results available (Q14, *N* = 114 responses). The majority of participants suggested induction treatment with intravenous methylprednisolone (IVMP) for 5 days (20–30 mg/kg/day, max. 1000 mg), followed by oral prednisolone (starting with 2 mg/kg/day, max. 100 mg/day) (93.86%). This was followed by IV cyclophosphamide (500–750 mg/m^2^ IV every 4 weeks for 4–6 months) (62.28%), MMF induction treatment (900–1200 mg/m2/day) (10.53%), and induction with oral prednisolone (2 mg/kg/day, max. 100 mg/day), followed by oral prednisolone in weaning dose (8/114). Both oral cyclophosphamide and azathioprine (1.5–2.5 mg/kg/day) were considered by (4/114) of responders (Table 3).

**Table 3:** Approach to treatment in sv-cPACNS.

**What medication would you give the patient? (Q14)**			
1.	Induction with IV methylprednisolone (IVMP) for 5 days (20–30 mg/kg/day, max. 1000 mg), followed by oral prednisolone starting with 2 mg/kg/day, max. 100 mg/day	107/114	93.86%
2.	IV Cyclophosphamide (500–750 mg/m2 every 4 weeks for 4–6 months)	71/114	62.28%
3.	Mycophenolate Mofetil (MMF) induction treatment (900–1200 mg/m2/day)	12/114	10.53%
4.	Induction with oral prednisolone (2 mg/kg/day, max. 100 mg/day), followed by oral prednisolone in creeping dose	8/114	7.02%
5.	Azathioprine (1.5–2.5 mg/kg/day)	4/114	3.51%
6.	Oral cyclophosphamide according to Fauci scheme	4/114	3.51%
7.	Others, Some respondents suggested a combination of steroid induction treatment along with IVMP while others suggested pulse IV cyclophosphamide. Two respondents suggested using MMF and Rituxab	18/114	15.79%
**Which anticoagulation would you initially choose? (Q15)**			
1.	IV heparin (100–150 units/kg/day)	59/112	52.68%
2.	Aspirin	25/112	22.32%
3.	None	17/112	15.18%
4.	Aspirin and clopidogrel in combination	4/112	3.57%
5.	Warfarin	3/112	2.68%
6.	Direct oral anticoagulants (DOACs), e.g., apixaban, rivaroxaban, betrixaban	0/112	0.00%
7.	Clopidogrel	0/112	0.00%
8.	Others, Most respondents who selected other indicated the use of low molecular weight heparin while others suggested the use of antiplatelet or anticoagulation medication in the treatment of cPACNS has been controversial with another saying heparin should be used but only in the case of an additional thrombotic event.	17/112	15.18%
**Which post-acute anticoagulation treatment would you consider? (Q16)**			
1.	Aspirin	53/107	49.53%
2.	None	16/107	14.95%
3.	Heparin SC	13/107	12.15%
4.	Warfarin	11/107	10.28%
5.	Aspirin and clopidogrel in combination	11/107	10.28%
6.	Direct oral anticoagulants (DOACs), e.g., apixaban, rivaroxaban, betrixaban	7/107	6.54%
7.	Others, one respondent said choice of treatment was dependent on the extent of infarction/haemorrhagic, while others were unsure and said they would seek further consultation from anticoagulation team and haematologists	8/107	7.48%
**Which immune modulating maintenance treatment would you consider? (Q17)**			
1.	Mycophenolate Mofetil (MMF: 900–1200 mg/m2/day)	75/113	66.37%
2.	Oral Prednisolone	27/113	23.89%
3.	Azathioprine (1.5–2.5 mg/kg/day)	26/113	23.01%
4.	IV Cyclophosphamide	20/113	17.70%
5.	Rituximab (375 mg/m2 four times, repeat as needed)	20/113	17.70%
6.	Methotrexate (10–20 mg/m2/Week)	11/113	9.73%
7.	TNF Blocker (Infliximab, Adalimumab, etc.)	5/113	4.42%
8.	Oral cyclophosphamide according to Fauci scheme	3/117	2.65%
9.	None	0/113	0.00%
10.	Others, respondents said they would consider a range of the above depending on what we thought underlying aetiology was, while others suggested steroids and rituximab.	6/113	5.31%
**How long would immune modulating maintenance treatment be required for in your opinion? (Q18)**			
1.	24 Months	39/114	34.21%
2.	12 Months	27/114	23.68%
3.	18 Months	20/114	17.54%
4.	36 Months	7/114	6.14%
5.	6 Months	4/114	3.51%
6.	Not necessary	1/114	0.88%
7.	3 Months	0/114	0.00%
8.	Others, Most respondents suggested a minimum of 12 months depending on imaging results, or after clinical remission. However, this is dependent on the course.	16/114	14.04%

Participants were then asked which anticoagulation treatment they would initially choose (Q15, *N* = 112 responses). The majority of respondents selected anticoagulation treatment with IV heparin (100–150 units/kg/day) (52.68%), followed by aspirin (22.32%), Aspirin and clopidogrel in combination (4/112), and warfarin (3/112). Notably, 15.18% suggested no anticoagulation treatment was required. Considering anticoagulation in the post-acute phase (Q16, *N* = 107 responses), acetylsalicylic acid (ASA) was the most frequently selected answer (49.53%), followed by subcutaneous (SC) heparin (12.15%), warfarin (10.28%), ASA and clopidogrel in combination (10.28%), and direct oral anticoagulants (DOACs). Notably, 14.95% (16/107) suggested that no anticoagulation treatment was required in the post-acute phase. When asked how long participants would continue anticoagulation (Q20, *N* = 106 responses), responses varied greatly with 12 months being the most selected answer (19.81%), followed by 6 and 24 months (17.92% each) (Table 3).

Next, immunomodulatory maintenance therapy was queried (Q17, *N* = 113 responses). The majority of respondents selected MMF (900–1200 mg/m2/day) (66.37%), followed by oral prednisolone (23.89%), azathioprine (1.5–2.5 mg/kg/day) (23%), IV cyclophosphamide (17.7%), rituximab (375 mg/m^2^ four times, repeat as needed) (17.7%), methotrexate (10–20 mg/m^2^/week) (9.73%), TNF inhibitors (Infliximab, Adalimumab, etc.; 5/113), and oral cyclophosphamide (3/113). All participants agreed that immunomodulatory maintenance was required. Considering treatment duration (Q18, *N* = 114 responses), most participants answered 24 months (34.21%), followed by 12 (23.68%), 18 (17.54%), 36 (7/114), and 6 months (4/113). None of the participants chose a short immunomodulatory maintenance course of 3 months. Notably, one participant suggested this form of treatment was unnecessary (Table 3). Respondents were also asked how long they would continue oral corticosteroid treatment (including tapering period) (Q19, *N* = 110 responses). A total of 43/110 (39%) suggested 6 months, 24 (21.82%) suggested 3 or 6 months, 4 suggested 18 months, 3 suggested 24, and 1 suggested 36 months with all participants indicating treatment with oral corticosteroids was necessary (Table 4).

**Table 4:** Therapeutic approach continued.

**How long would you give oral corticosteroids treatment for (including slow taper)? (Q19)**			
1.	6 Months	43/110	39.09%
2.	12 Months	24/110	21.82%
3.	3 Months	24/110	21.82%
4.	18 Months	4/110	3.64%
5.	24 Months	3/110	2.73%
6.	36 Months	1/110	0.91%
7.	Not necessary	0/110	0.00%
8.	Others, Most respondents indicated this depends on course and imaging and markers, but at least 6–12.	11/110	10.00%
**When would you discontinue anticoagulation treatment? (Q20)**			
1.	12 Months	21/106	19.81%
2.	24 Months	19/106	17.92%
3.	6 Months	19/106	17.92%
4.	No need for anticoagulation	15/106	14.15%
5.	3 Months	8/106	7.55%
6.	36 Months	4/106	3.77%
7.	18 Months	3/106	1.83%
8.	Others, Respondents indicated that this is again heavily dependent on the course and MRI findings but most respondents suggested at least 2 years.	17/106	16.04%
**When would you repeat MRI? (Q21)**			
1.	3 Months	76/111	68.47%
2.	6 Months	54/111	48.65%
3.	12 Months	39/111	35.14%
4.	24 Months	25/111	22.52%
5.	36 Months	13/111	11.71%
6.	18 Months	10/111	9.01%
7.	unnecessary	4/111	3.60%
8.	Others, Respondents indicated that depending on clinical status and response to treatment a check MRI can be performed after 1 month with subsequent checks favoured as well.	16/111	14.41%
**When would you want a clinical follow up? (Q22)**			
1.	3 Months	89/111	80.18%
2.	6 Months	33/111	29.73%
3.	12 Months	30/111	27.03%
4.	24 Months	28/111	25.23%
5.	18 Months	25/111	22.52%
6.	36 Months	23/111	20.72%
7.	None	0/111	0.00%
8.	Others, Responses again varied with the general consensus being weekly initially, followed by monthly and then every 3 months when the patient has stabilised.	31/111	27.93%
**2 years later, the patient develops a disease flare up. What immune modulating medication would you use to treat this? (Q23)**			
1.	Intravenous methylprednisolone (IVMP)	83/112	74.11%
2.	Rituximab (375 mg/m2 four times, repeat if necessary)	46/112	41.07%
3.	Mycophenolate Mofetil (MMF) induction treatment (900–1200 mg/m2/day)	32/112	28.57%
4.	IV Cyclophosphamide	31/112	27.68%
5.	Oral prednisolone	20/112	17.86%
6.	TNF Blocker (Infliximab, Adalimumab, etc.)	13/112	11.61%
7.	Methotrexate (10–20 mg/m2/week)	7/112	6.25%
8.	Azathioprine (1.5–2.5 mg/kg/day)/azathioprine (1.5–2.5 mg/kg/day)	5/112	4.46%
9.	Oral cyclophosphamide according to Fauci scheme	1/112	0.89%
10.	None	0/112	0.00%
11.	Others, Respondents indicated that this depends on what the patient responded to initially, with others suggesting a mixture of IVMP, TNF blockers, MMF as well as rituximab.	17/112	15.18%

### Disease Monitoring and Flare Management

**Figure 2:**
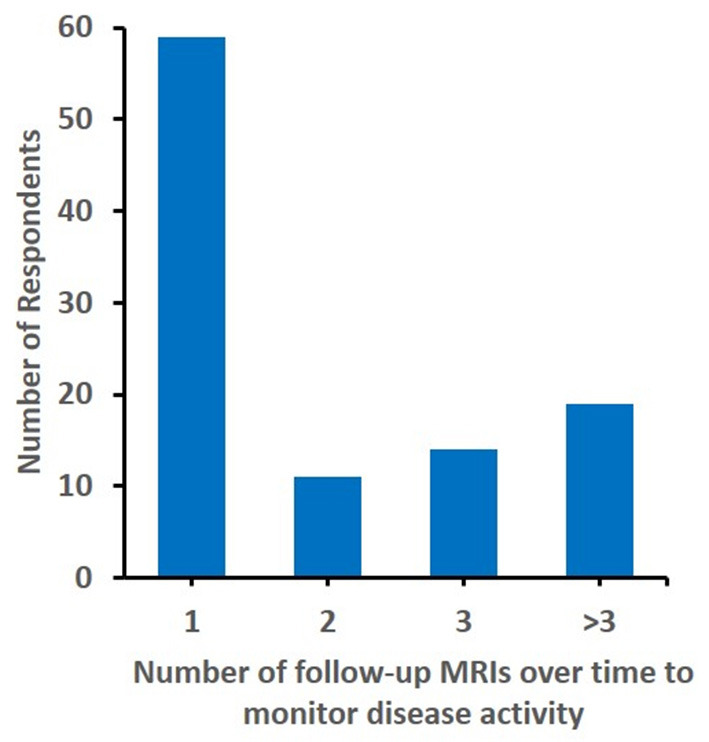
Number of follow up MRIs considered. The number of follow-up MRIs to montior diseadse activity considered by (*N* = 103) responents is displyed.

To get an understanding of treatment and response monitoring chosen, participants were asked when they would repeat MRI (Q21, *N* = 111 responses). At 3 months, 68.47% would repeat MRI. With increasing time from diagnosis, fewer colleagues would perform MRI: at 6 months 48.65%, at 12 months 35.14%, at 24 months 22.52%, at 36 months 11.71%, and at 18 months 9.01%. Interestingly, 4 participants suggested that a repeat MRI was unnecessary, and a large proportion (57.28%) would only perform one follow-up MRI (Figure 2). Considering clinical disease monitoring (Q22, *N* = 111 responses), a large proportion of respondents (80.18%) would schedule clinical follow-up at 3 months, followed 29.73% at 6, 27% at 12, 25.23% at 24, 22.5% at 18, and 20.72% at 36 months.

Next, participants were informed that 2 years after diagnosis, the patient develops a disease flare and asked what immune modulating medication they would consider (Q23, *N* = 112 responses). The majority of respondents selected IVMP as the treatment of choice (74.11%), followed by rituximab (41.07%), MMF induction treatment (28.57%), IV cyclophosphamide (27.68%), oral prednisolone (17.86%), TNF inhibitors (11.61%), methotrexate (7/112), azathioprine (5/112), one respondent suggested oral cyclophosphamide (Table 4).

### Medical Specialities Involved

Participants were asked which medical sub-specialities should be involved in the treatment of the patient presented (Q24, *N* = 113 responses). As multiple options could be selected by participants the following responses are the accumulative response of each option. Responses included Paediatric Neurology (99.12%), followed by Rheumatology (89.38%), Radiology (83.16%), Intensive care medicine (63.72%), Haematology (46.90%), Infectious diseases (42.48%), Oncology (24.78%) with all specialities mentioned (21.24%) (Table 5).

**Table 5:** Specialities involved in the treatment of sv-cPACNS.

**What Specialities do you believe should be involved in the treatment of the Case study? (Q24)**			
1.	Neuropediatrics	112/113	99.12%
2.	Rheumatology	101/112	89.38%
3.	Radiology	94/112	83.16%
4.	Intensive care	72/112	63.72%
5.	Haematology	53/112	46.90%
6.	Infectious diseases	48/112	42.48%
7.	Oncology	28/112	24.78%
8.	All mentioned	24/112	21.24%
9.	Others, Respondents that selected other suggested that haematologists, immunologist, Neurologist as well as neurosurgeons may all be important in the treatment of this patient.	17/112	15.04%

### Influence of Experience

**Figure 3:**
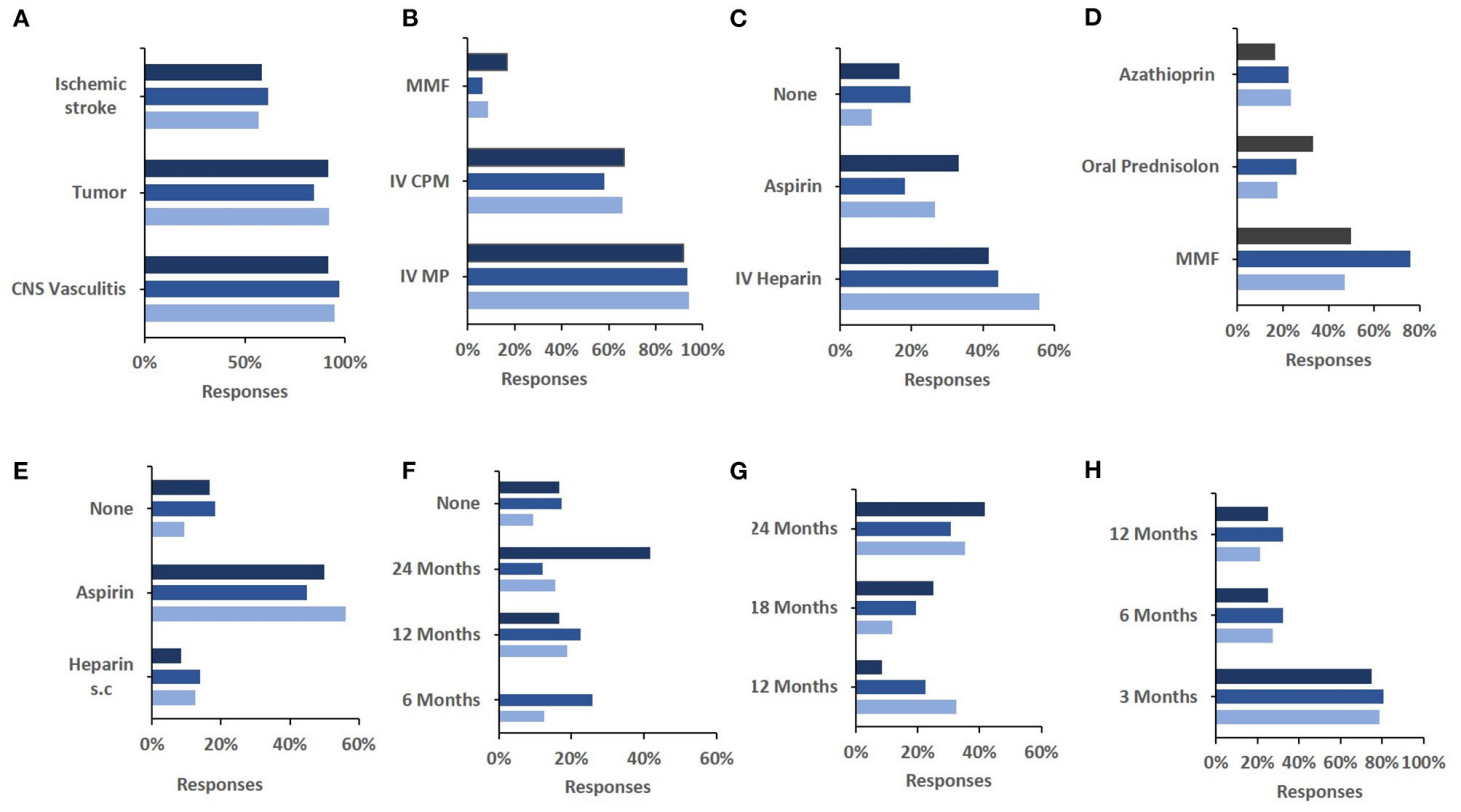
Influence of experience of patients treated in their approach to diagnosis and treatment of the case study. **(A)** The correlation between number of sv-cPACNS patients treated and their differential diagnosis of the case study patient (*N* = 12, 65, 37). **(B)** The number of sv-cPACNS patients treated agains induction treatment chosen (*N* = 12, 62, 35). **(C)** The number of sv-cPACNS patients treated agains acute anticoagulation treatment (*N* = 12, 61, 34). **(D)** The number of sv-cPACNS patients treated agains maintenance treatment (*N* = 12, 62, 34). **(E)** The correlation between number of sv-cPACNS patients treated agains post acute anticoagulation maintenance treatment they would consider (*N* = 12, 60, 32). **(F)** The number of sv-cPACNS patients treated agains duration of immune modulating maintenance treatment (*N* = 12, 62, 34). **(G)** The number of sv-cPACNS patients treated vs. length of discontinuation of anticoagulation maintenance treatment (*N* = 12, 58, 32). **(H)** The number of sv-cPACNS patients treated Vs. length of follow up (*N* = 12, 62, 33). MMF, myophelolate mofetil; ASA, acetylsalicylic acid.

Next, we also aimed to assess whether personal professional experience affects the approach to diagnosis and treatment of sv-cPACNS (Q4, *N* = 141 responses). Due to sometimes varied responses to individual questions and little differences between answers less frequently chosen, we decided to only look at top three responses. Overall, no marked differences were seen between less and more experienced colleagues in relation to differential diagnoses considered (Figure 3A), acute phase (Figures 3B,C) or maintenance (Figure 3D) treatment.

This can similarly be seen for anticoagulation maintenance treatment (Figure 3E), duration of maintenance treatment (Figure 3F), and length of follow up (Figure 3G). However, the same cannot be said for the length of discontinuation of anticoagulation maintenance treatment (Figure 3H), as responses varied considerably with respondents with >5 years' experience heavily favouring 24 months before ceasing treatment. This can be seen as the reverse of respondents with ≤ 5 years' experience with 24 months being there least selected option. These respondents indicated that 6 months is sufficient.

### Regional Differences Between Europe and North America

**Figure 4:**
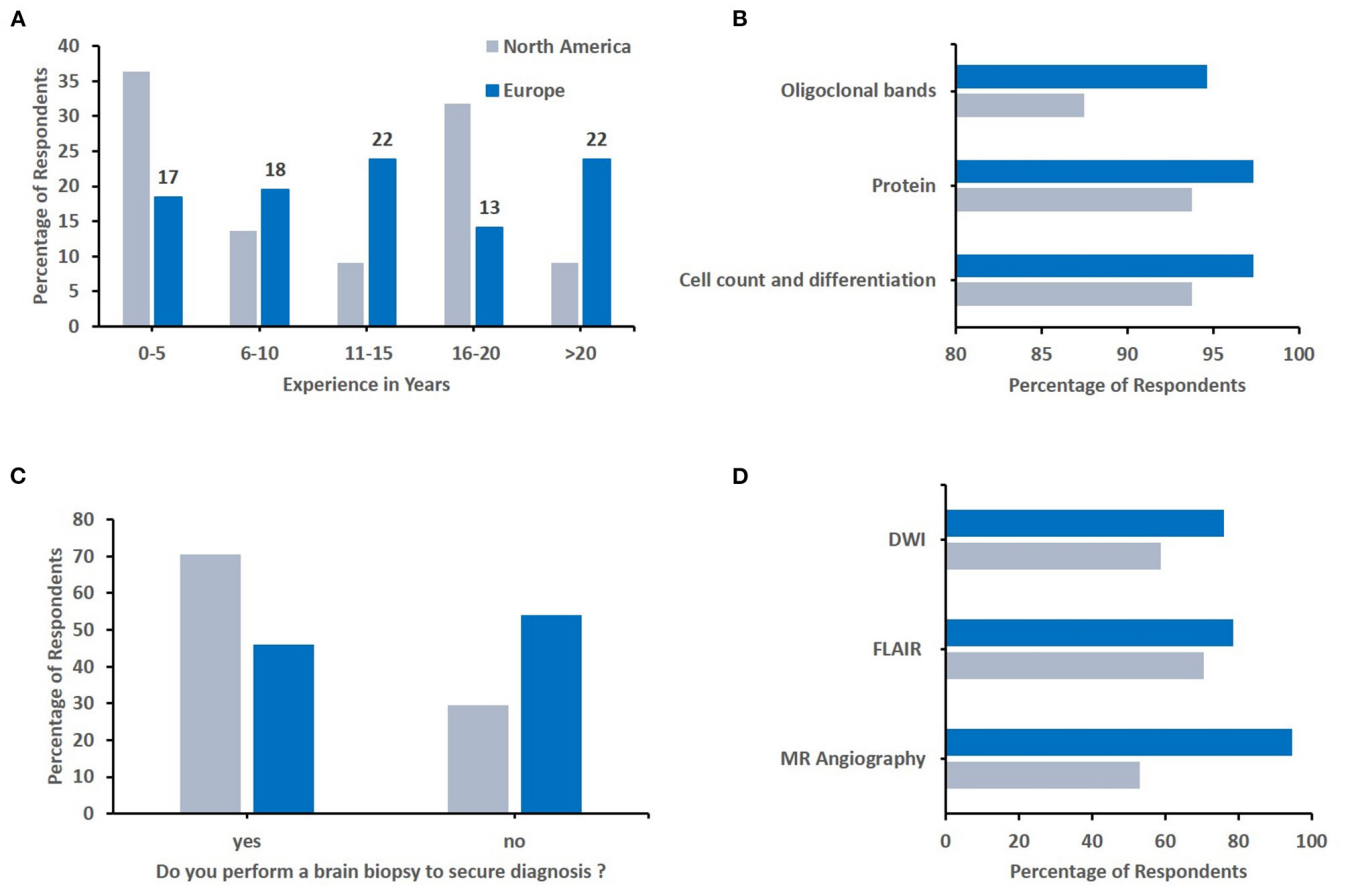
Geographical differences in diagnostic approaches. Responses were compared between responddents from Europe and North America. **(A)** Number of years respondents have worked in their speciality (North America *N* = 22, Europe *N* = 92). **(B)** Key parameters respondents would determine from cerebrospinal fluid (North America *N* = 16, Europe *N* = 75). **(C)** The percentage of respondents who would/wouldn't perform a brain biopsy to secure diagnosis (North America *N* = 17, Europe *N* = 74). **(D)** Sequences of the emergency MRI chosen by respondents (North America *N* = 17, Europe *N* = 75).

**Figure 5:**
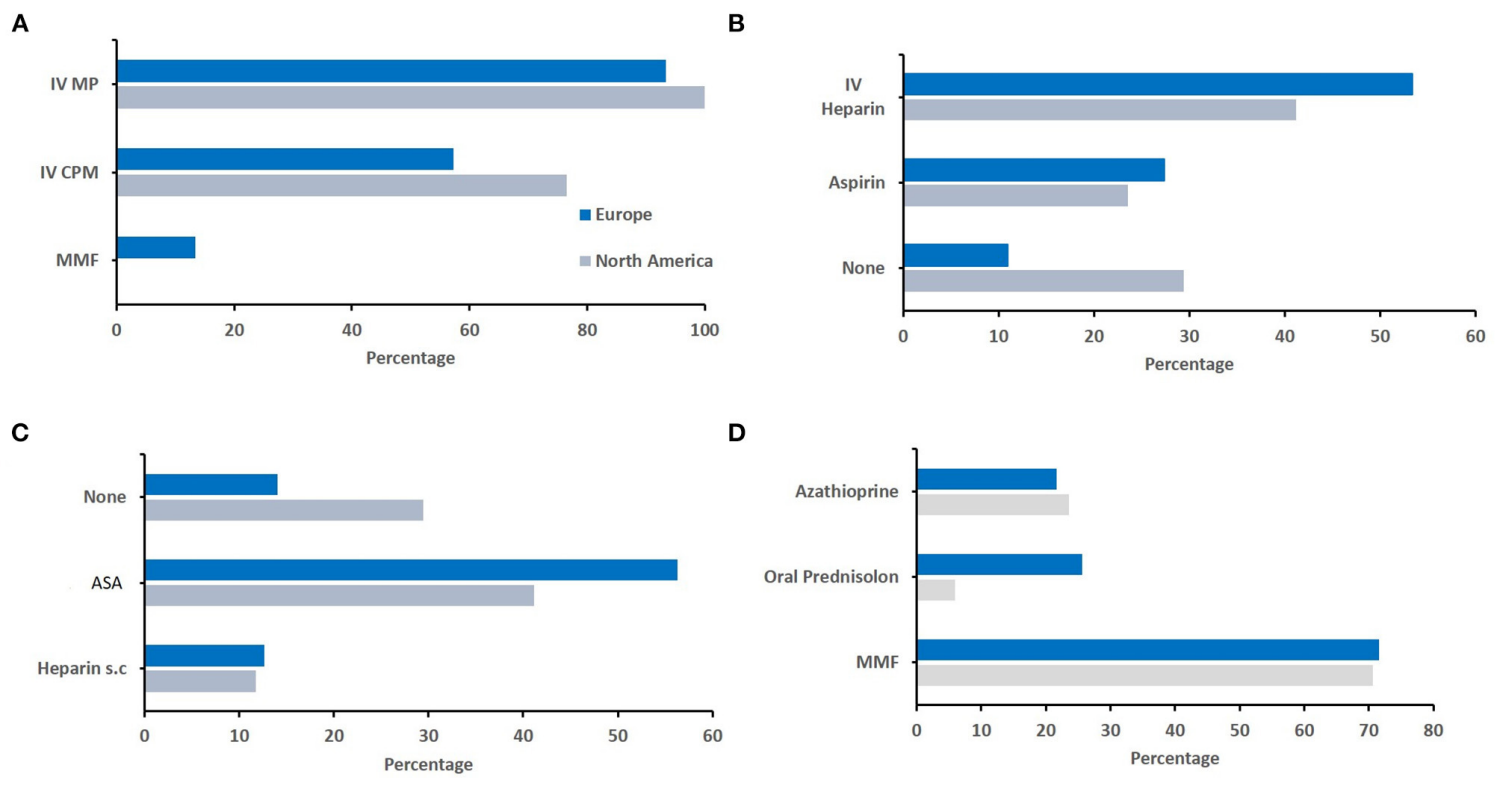
Geographical differences in therapeutic approaches. Responses were compared between responddents from Europe and North America. **(A)** Immunomodulating induction treatment (North America *N* = 17, Europe *N* = 75). **(B)** Acute anticoagulation treatment (North America *N* = 17, Europe *N* = 73). **(C)** Post-acute anticoagulation maintenance treatment (North America *N* = 17, Europe *N* = 71). **(D)** Immunomodulating maintanence treatment (North America *N* = 17, Europe *N* = 74).

Finally, we aimed to assess whether differences in relation to diagnosis and treatment of sv-cPACNS exist between geographic regions (Q4, *N* = 141 responses). As most responses were recorded from these regions, we focused our efforts to North America (*N* = 22) and Europe (*N* = 92) (Figure 4). As agreement was highest among top choices, and individual responses varied among less frequently chosen options, the top three responses are reported here. The proportion of more experienced colleagues (in years) was slightly higher in Europe (Figure 4A), and oligoclonal bands and MRI angiography were more frequently considered by Europeans (Figures 4B,D). Notably, North American colleagues more commonly considered brain biopsied to secure diagnosis (Figure 4C). Considering treatment, notable differences existed in the choice of induction treatment. In Europe, respondents more frequently chose IVMP followed by MMF or azathioprine and oral prednisolone tapering schemes, while North American colleagues more frequently included cyclophosmamide in their treatment induction regimen (Figure 5).

## Discussion

Small vessel (sv-)cPACNS is a rare immune-mediated inflammatory disease with variable clinical presentation, including headaches and fatigue, seizures, psychiatric symptoms, neurological deficits, and others. Treatment aims at the prevention of (further) damage and is based on individual case reports, small case series, expert opinion, and personal experience, and includes anti-inflammatory agents. As ongoing inflammation, vessel occlusion and resulting ischemia can cause significant damage and even be lethal, timely and correct diagnosis and treatment initiation are of utmost importance (11, 21). Based on reports in the literature, sv-cPACNS is frequently considered to be more aggressive when compared to medium to large vessel p-cPACNS. Thus, some authors use more aggressive treatment regimens for sv-PACNS, including the cytotoxic agent cyclophosphamide (22). Due to the lack of widely accepted and prospectively evaluated diagnostic criteria, treatment recommendations and clinical trials, clinical practise varies significantly between centres and geographical regions (17).

In rare but severe conditions, in which clinical trials are hard/impossible to achieve, consensus treatment plans can be developed as a tool to at least partially overcome aforementioned challenges, aiming at harmonisation of diagnostic and therapeutic approaches to improve quality of care and outcomes. A first step towards the development of such plans is the collection of information about the current standard of care delivered by physicians specialised in the care of patients with rare diseases (18, 23, 24). This manuscript reports results from an international online survey collecting data on diagnostic and therapeutic approaches currently used in patients with sv-cPACNS. A relatively low overall response rate of 1.43% may likely be explained by limited experience across specialties and countries due to the rare nature of the disease. Indeed, a majority of responses appear to be from experienced specialists with experience treating sv-cPACNS patients, as only 19.29% of respondents had <5 years' experience as a specialist, and >66% had personal experience treating patients with cPACNS.

Overall, consensus existed among respondents on diagnostic approaches, including routine blood tests (including full blood counts, inflammatory markers and clotting tests), emergency MRI of the brain including angio-MRI, and CSF analysis during acute presentation. Indeed, reports in the literature suggest that up to 50% of patients with sv-cPACNS display signs of systemic inflammation, MRI anomalies including increased signal on T2-weighted sequences, and pathological findings on CSF exams, such as elevated proteins and pleocytosis (4, 12, 22). To exclude differential diagnoses, including systemic vasculitis or other neuroinflammatory disease (25), blood (antiphospholipid antibodies, ANA, ANCA, etc) and CSF (Aquaporin and Anti-NMDA) immunology tests were suggested by a majority (4, 12, 22).

As CSF and blood anomalies in the absence of autoantibodies are not specific findings in favour of cPACNS, MRI imaging and brain biopsies are considered valuable tools (26). This was reflected by answers from a majority of respondents who use emergency MRI of the brain including angio-MRI in patients with new neurological deficits. Most respondents suggested using MR angiography (86.84%), FLAIR (74.56%) and diffusion weighted MRI sequences (66.67%), followed by T1 FS, contract enhanced (54.39%) as well as T2 FS (46.49%). This is in agreement with the literature, as in a cohort of 66 children diagnosed with cPACNS, MRI lesions were best seen using a combination of FLAIR/T2 sequences when compared to DWI alone (26). Another study found that roughly half of patients presented with normal head CT scans while 90% had abnormal brain MRI (12). Interestingly, only 83% of respondents considered radiologists as essential team members in the care of sv-cPACNS patients.

While criteria for the diagnosis of sv-PACNS (7, 8) require a brain biopsy (16), only 46.09% of respondents indicated that they would perform this procedure. Especially in cases without MRI anomalies suggesting localisation of inflammatory changes or alterations in regions not accessible to biopsies, colleagues were cautions, and some colleagues hesitated to perform biopsies (53.04%) with some insinuating it is a too invasive procedure. This is in partial disagreement with reports from the literature that suggest brain biopsies should target accessible lesions identified by the MRI, however, if lesions are not easily accessible, non-lesional biopsy's may be performed that target the non-dominant frontal lobe (12, 19, 27). Interestingly, when comparing differences in diagnostic and therapeutic approaches between Europe and North America, p higher proportion of North American as compared to European respondents would perform a brain biopsy to secure diagnosis (70.59 vs. 45.95%). While the reasons remain speculation, this may be in part be due to current criteria for sv-cPACNS, which include evidence of vasculitis on brain biopsy), originating from North America (8).

Therapeutic approaches are the subject of intense discussions as randomised and controlled clinical trials are absent for sv-cPACNS. In this survey, nearly all respondents prefer induction treatment with IVMP pulses for 5 days, followed by an oral prednisolone tapering scheme (93.86%). A smaller proportion of respondents favoured or included IV cyclophosphamide as induction treatment (62.28%). Notably, induction treatment with IV cyclophosphamide was more commonly suggested by North American when compared with European respondents (76.47 vs. 57.33%). Previously, cyclophosphamide treatment was tested in an open label study including 19 sv-cPACNS patients, 14 of whom completed induction therapy and moved to maintenance treatment. However, eight patients experienced disease flares, two already during induction and five during maintenance treatment with another experiencing a late disease flare, off medication. Notably, no patients relapsed on maintenance treatment with MMF (12). As no control cohort was involved, this study only suggests some level of efficacy mediated by cyclophosphamide while not offering comparisons to other (less toxic) drugs. Another study treated 25 sv-cPACNS patients with high-dose prednisone including a 12-month taper, 20 patients received IV cyclophosphamide for 6 months along with either of MMF/azathioprine, while 3 patients received only MMF/azathioprine as induction/maintenance treatment. Two patients received no treatment. They reported that disease activity was increased at the time of diagnosis but across all groups significantly decreased with treatment over time. Six patients had a disease flare up with 52% having a good neurologic outcome after 12 months (22). As most published reports report response to monthly infusions of cyclophosphamide for induction treatment of sv-cPACNS but concerns in relation to necessity in all patients exist, it was interesting to see that “only” 62% of respondents consider this cytotoxic agent to induce remission. However, this may reflect developments in other severe autoimmune/inflammatory diseases, in which cyclophosphamide is gradually replaced by other, less toxic, therapeutic agents (20).

For anti-inflammatory maintenance treatment, MMF was favoured by the majority of respondents (66.37%), followed by long-term oral prednisolone (23.89%). Post-acute phase corticosteroid tapering regimens (to bridge from induction treatment to efficacy of maintenance therapy) vary across specialists, centres and disorders treated (16). Most colleagues responding to this survey would prescribe oral corticosteroids for 6 months (39.09%), closely followed by 12 (21.82%) and 3 (21.82%) months. Duration of maintenance immunomodulating treatment from participants ranged from 24 months (34.21%), 12 months (23.68%) and then closely followed by 18 months (17.54%) with respondents suggesting that maintenance treatment be for a minimum of 12 months. This is agreement with reports in the literature indicating that MMF for 18 months as maintenance therapy. Hutchinson et al. (12) reported that this maintenance therapy should be favoured over azathioprine to avoid the potential for treatment failure/intolerance (16).

Anticoagulation treatment appears to be inconsistent between centres; 52.68% of participants use IV heparin in the acute phase, followed by aspirin with just over 1/7 of respondents suggesting they would not treat with anticoagulation treatment. Aspirin was then the most favoured anticoagulation treatment during the post actuation phase, with the next selected response being no anticoagulation treatment again. Significant uncertainty also exists around the question of how long to continue antiplatelet treatment with ASA with most colleagues treating for 12 months (19.81%). Interestingly the available literature indicates that low molecular weight IV heparin, aspirin, and warfarin are the most frequently used anticoagulants in p-cPACNS; for sv-cPACNS no published data exists. The length of anticoagulation treatment in p-cPACNS also differs throughout the literature (28). A scoping review of cPACNS found that treatment with anticoagulants is not commonly seen in sv-cPACNS (16).

Considerable agreement exists in relation to clinical and MRI monitoring, as most participants consider MRI screening at 3, 6, and 12 months with clinical follow up at 3 months.

Therapeutic uncertainty in this rare condition was further underscored when participants were confronted with disease flares, in which IVMP and rituximab were most frequently chosen. To our knowledge, no published evidence exists for the use of rituximab in sv-cPACNS. Also, other biopharmaceutic agents may be an option, such as TNF inhibitors, but were not considered by respondents to this survey, which may be the result of very limited evidence for their use and efficacy in sv-cPACNS (29).

While this survey provides insight into diagnostic and therapeutic approaches to sv-cPACNS collected from a sizable cohort of international experts, it is limited by the aforementioned relatively small number of responses that, however, may also suggest that primarily colleagues experienced with or interested in sv-cPACNS participated. A total of 47 participants had no personal experience which, however, did not affect responses when compared to colleagues experienced in the treatment of sv-cPACNS (Figures 2, 3).

## Conclusions

This survey collected information around diagnostic and therapeutic approaches in sv-PACNS. While approaches were mostly consistent across respondents, uncertainty exists around the role of brain biopsies to diagnose sv-cPACNS, the role of cyclophosphamide during induction treatment, and anticoagulation to prevent ischemia. Prospective and systematic collection of data in international collaborations is necessary to improve the evidence base and work towards informed diagnostic and treatment recommendations.

## Data Availability Statement

The raw data supporting the conclusions of this article will be made available by the authors, without undue reservation.

## Ethics Statement

Ethical review and approval was not required for the study on human participants in accordance with the local legislation and institutional requirements. Written informed consent for participation was not required for this study in accordance with the national legislation and the institutional requirements.

## Author Contributions

PK, JB, and CH lead on data analysis and preparation of the first written draught. All authors were involved in study planning, manuscript preparation, and read and agreed on the final version of this manuscript.

## ACKNOWLEDGEMENTS

The authors thank Jana Hörstermann, Deutsches Rheuma-Forschungszentrum, Berlin, Germany for support in preparing and executing the Survey.

## Conflict of Interest

The authors declare that the research was conducted in the absence of any commercial or financial relationships that could be construed as a potential conflict of interest.

## Publisher's Note

All claims expressed in this article are solely those of the authors and do not necessarily represent those of their affiliated organizations, or those of the publisher, the editors and the reviewers. Any product that may be evaluated in this article, or claim that may be made by its manufacturer, is not guaranteed or endorsed by the publisher.
