# Supplementary material for: Diagnosis and Treatment of Small Vessel Childhood Primary Angiitis of the Central Nervous System (sv-cPACNS): An International Survey
Source: Front Pediatr. 2021 Oct 12;9:756612. doi: 10.3389/fped.2021.756612 (PMC8546335; doi:10.3389/fped.2021.756612)
Supplement: Supplementary file 1 [file Data_Sheet_1.DOCX]

**Appendix**

**Survey**

**Introductory Questions:**

**Q 1.What is your (sub-)specialty? E.g., Paediatric Rheumatology, Neurology, General Paediatrics, Radiology, etc.**

**Q 2. How many years’ experience have you had in this specialty? i.e. as a fellow, consultant/attending**

**Q 3. What country are you currently working in?**

**Q 4. Have you treated patients with small vessel primary angiitis of the central nervous system (sv-cPACNS)?**

Y/N

If yes, how many?

**A clinical scenario will be outlined, and you will be asked to answer questions on how you would systematically approach the patient and the treatment you would give.**

***Case Study***

***A previously healthy 10-year-old girl came to hospital with an approximately one month-long history of headaches, nausea, and gradual hemiparesis on her left. She had been admitted six weeks previously with left-sided focal seizures.***

**Q 5. Do you believe genetic testing plays a role in diagnosing sv-cPACNS (small vessel childhood primary angiitis of the central nervous system)?**

If yes, what genes would be of interest to you? (please state)

**Q 6. Based on the patient’s history, what are the most important differentials to consider? Choose the top 5.**

* Migraine

* Infection, i.e. Meningitis

* Congenital deformity

* CNS vasculitis

* Sub-acute degeneration of the spinal cord (vitamin D deficiency)

* Central nervous system Tuberculosis (CNS TB)

* Tumour

* Traumatic haemorrhage

* Ischemic stroke

* Multiple Sclerosis

* Others (please state)

**Q 7. What first-line investigations would you perform?**

* Blood Tests (including full blood counts, inflammatory markers and clotting tests)

* Lumbar Puncture and CSF analysis, including cell counts and differentiation, protein, lactate, glucose, microbial cultures, herpes virus PCR, VZV PCR, Borrelia IgG, IgM

* Emergency MRI of the brain including angio-MRI

* Brain CT scan including CT angiography

* All of the above

* None

* Others (please state)

**Q 8. If you chose blood tests, which of the following bloods would you order?**

* N/A

* Full Blood Count (including complete white cell count)

* Clotting tests (including PTT, INR, fibrinogen, D dimers)

* Interferon Gamma Release Assay Test (for TB infection)

* Immunology (e.g. ANA, ENA, Complement Factors, Cardiolipin AB, ANCA)

* Adenosine deaminase 2 activity (ADA2)

* All of the above

* None

* Others (please state)

**Q 9. If you chose to look at blood immunology, which of the following would you look at?**

* N/A.

* Antinuclear Antibodies (ANA)

* Anti-double stranded DNA (dsDNA)

* Complement factors and complement cascade activation

* Anti-phospholipid Antibodies

* Anti-neutrophil Cytoplasmic Antibody (ANCA)

* Anti-NMDA and aquaporin antibodies

* All of the above

* None

* Others (please state)

**Q 10. If you chose lumbar puncture, which of the following would you look at?**

* N/A

* LP opening pressure

* Cell count and differentiation

* Protein

* Lactate

* Oligoclonal Bands

* Anti-NMDA and aquaporin antibodies

* Glucose

* Culture

* All of the above

* None

* Others (please state)

**Q 11. If you chose (Emergency*) MRI, which of the following are you interested in?**

*ASAP, same day

* N/A

* Diffusion-weighted MRI (DWI)

* T1 with fat saturation (FS)

* T1 FS, contract enhanced

* T2 FS

* TIRM (Turbo inversion recovery magnitude)/STIR (Short tau inversion recovery)

* FLAIR (Fluid-attenuated inversion recovery)

* MR Angiography

* All of the above

* None

* Other (please state)

***Cerebrospinal fluid opening pressure, cell count, cytology, protein and glycose concentrations were normal, and cultures and HSV polymerase chain reaction were negative. On brain MRI (FLAIR sequences), signal alterations in the parietal region were identified. T1 gadolinium enhances sequences unveiled enhancement in the affected region.***


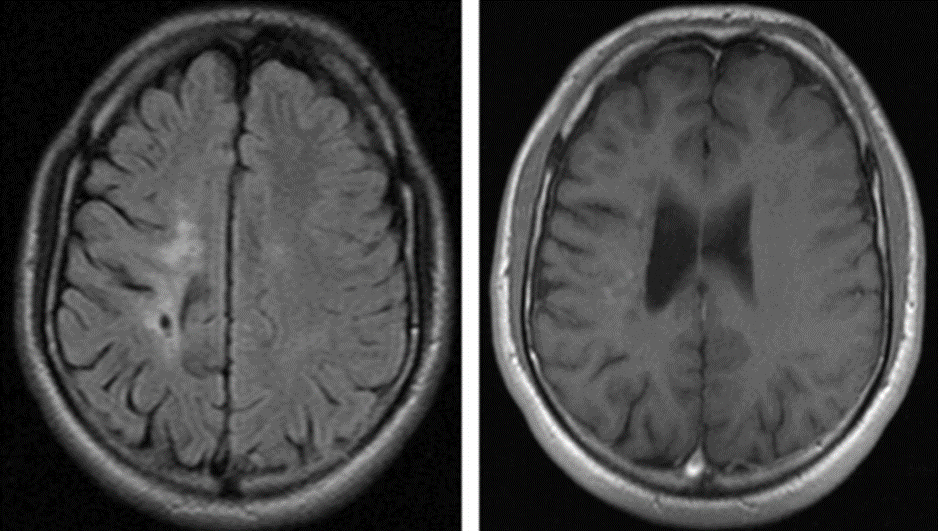


**Q 12. The diagnosis suspected small vessel vasculitis is made. Would you perform a brain biopsy to confirm the diagnosis?**

* Yes

* No (what else would you do? Please state)

**Q 13. In which of these situations would you perform a brain biopsy?**

* Otherwise stable patient with slowly progressing hemiplegia and aforementioned MRI findings

* Patient on ICU in status epilepticus, ventilated, with aforementioned MRI, and lesions are accessible to biopsy

* Patient in ICU in status epilepticus, ventilated, with aforementioned MRI, and lesions not accessible for biopsy (-> biopsy from non-lesional tissue)

* Patient on ICU in status epilepticus, ventilated, with no findings on MRI

* Under all the aforementioned circumstances

* Never

Brain biopsy shows a small vessel, non-granulomatous, non-necrotic, lymphocytic angiitis.

You have made the diagnosis of primary small vessel vasculitis based on clinical findings, lab and MRI findings, plus suggestive biopsy.

**Q 14. What medication would you give the patient?**

* Initiation with IV Methyprednisolone (IVMP) over 5 days (20-30mg/kg/day, up to 1000mg), followed by oral Prednisolone staring at 2 mg/kg/day, up to 100mg/day

* Initiation with oral Prednisolone (2 mg/kg/day, up to 100mg/day), followed by oral Prednisolone taper

* Intravenous Cyclophosphamide (500-750mg/m2 i.v. every 4 weeks for 4-6 months)

* Oral Cyclophosphamide following Fauci-Scheme

* Mycophenolate Mofetil (MMF) induction treatment (900-1200 mg/m2/day)

* Azathioprine (1.5-2.5mg/kg/day)

* Other (please state)

**Q 15. Which acute anticoagulation treatment would you consider?**

* None

* Heparin i.v. (100-150 units/kg/day)

* Aspirin

* Warfarin

* Clopidogrel

* Combination of Aspirin and Clopidogrel

* Direct Oral Anticoagulants (DOACs) e.g. Apixaban, Rivaroxaban, Betrixaban

* Other (please state)

**Q 16. Which post-acute anticoagulation treatment would you consider?**

* None

* Heparin s.c.

* Aspirin

* Warfarin

* Clopidogrel

* Combination of Aspirin and Clopidogrel

* Direct Oral Anticoagulants (DOACs) e.g. Apixaban, Rivaroxaban, Betrixaban

* Other (please state)

**Q 17. Which immune modulating maintenance treatment would you consider?**

* None

* Cyclophosphamide i.v.

* Oral Cyclophosphamide following Fauci scheme

* Mycophenolate Mofetil (MMF: 900-1200 mg/m2/day)

* Methotrexate (10-20mg/m2/week)

* Oral prednisolone

* Azathioprine (1.5-2.5mg/kg/day)

* Rituximab (375mg/m2 four times, repeat as needed)

* Anti-TNF agent (Infliximab, Adalimumab, etc.)

* Other (please state)

**Q 18. How long would immune modulating maintenance treatment be required for in your opinion?**

* Not necessary

* 3 months

* 6 months

* 12 months

* 18 months

* 24 months

* 36 months

* Other (please state)

**Q 19. How long would you give oral corticosteroids treatment for (including slow taper)?**

* Not necessary

* 3 months

* 6 months

* 12 months

* 18 months

* 24 months

* 36 months

* Other (please state)

**Q 20. When would you discontinue anticoagulation treatment?**

* Not necessary

* 3 months

* 6 months

* 12 months

* 18 months

* 24 months

* 36 months

* Other (please state)

**Q 21. When would you repeat MRI?**

* Not necessary

* 3 months

* 6 months

* 12 months

* 18 months

* 24 months

* 36 months

* Other (please state)

**Q 22.When would you want a clinical follow up?**

* 3 months

* 6 months

* 12 months

* 18 months

* 24 months

* 36 months

* Other (please state)

* None

**Q 23. 2 years later, the patient develops a disease flare up. What immune modulating medication would you use to treat this?**

* None

* Intravenous Methyprednisolone (IVMP)

* Oral prednisolone

* Cyclophosphamide i.v.

* Oral Cyclophosphamide following Fauci scheme

* Mycophenolate Mofetil (MMF) induction treatment (900-1200 mg/m2/day)

* Azathioprine (1.5-2.5mg/kg/day)

* Methotrexate (10-20mg/m2/week)

* Rituximab (375mg/m2 four times, repeat as needed)

* Anti-TNF agent (Infliximab, Adalimumab, etc.)

* Other (please state)

**Q 24. What Specialities do you believe should be involved in the treatment of Case 1? (Multiple Options)**

* Paedeatric Neurology

* Rheumatology

* Haemotology

* Radiology

* Oncology

* Intensive Care

* Infectious Diseases

* All of the Above

* Other (please state)
